# Budget impact analysis of chronic kidney disease mass screening test in Japan

**DOI:** 10.1007/s10157-014-0943-8

**Published:** 2014-02-11

**Authors:** Masahide Kondo, Kunihiro Yamagata, Shu-Ling Hoshi, Chie Saito, Koichi Asahi, Toshiki Moriyama, Kazuhiko Tsuruya, Tsuneo Konta, Shouichi Fujimoto, Ichiei Narita, Kenjiro Kimura, Kunitoshi Iseki, Tsuyoshi Watanabe

**Affiliations:** 1 Department of Health Care Policy and Health Economics, Faculty of Medicine, University of Tsukuba, 1-1-1 Tennoudai, Tsukuba, Ibaraki 305-8577 Japan; 2 Department of Nephrology, Faculty of Medicine, University of Tsukuba, 1-1-1 Tennoudai, Tsukuba, Ibaraki 305-8575 Japan; 3 Department of Chronic Kidney Disease Initiatives, Fukushima Medical University School of Medicine, 1 Hikarigaoka, Fukushima, Fukushima 960-1295 Japan; 4 Health Care Center, Osaka University, 1-17 Machikaneyama-cho, Toyonaka, Osaka 560-0043 Japan; 5 Department of Integrated Therapy for Chronic Kidney Disease, Graduate School of Medical Sciences, Kyushu University, 3-1-1 Maidashi, Higashi-ku, Fukuoka, Fukuoka 812-8582 Japan; 6 Department of Cardiology, Pulmonology, and Nephrology, Yamagata University School of Medicine, 2-2-2 Iida-Nishi, Yamagata, Yamagata 990-9585 Japan; 7 Department of Hemovascular Medicine and Artificial Organs, Faculty of Medicine, University of Miyazaki, 5200 Kihara, Kiyotake, Miyazaki, Miyazaki 889-1692 Japan; 8 Division of Clinical Nephrology and Rheumatology, Graduate School of Medical and Dental Sciences, Niigata University, 1-757 Chuo-ku, Niigata, Niigata 951-8510 Japan; 9 Division of Nephrology and Hypertension, Department of Internal Medicine, St. Marianna University School of Medicine, Sugao 2-16-1, Miyamae-Ku, Kawasaki City, Kanagawa 216-8511 Japan; 10 Dialysis Unit, University Hospital of The Ryukyus, 207 Uehara, Nishihara, Okinawa 903-0215 Japan; 11 Department of Nephrology, Hypertension, Diabetology, Endocrinology and Metabolism, Fukushima Medical University School of Medicine, 1 Hikarigaoka, Fukushima, Fukushima 960-1295 Japan

**Keywords:** CKD, Budget impact, Dipstick test, Mass screening, Proteinuria, Serum creatinine assay

## Abstract

**Background:**

Our recently published cost-effectiveness study on chronic kidney disease mass screening test in Japan evaluated the use of dipstick test, serum creatinine (Cr) assay or both in specific health checkup (SHC). Mandating the use of serum Cr assay additionally, or the continuation of current policy mandating dipstick test only was found cost-effective. This study aims to examine the affordability of previously suggested reforms.

**Methods:**

Budget impact analysis was conducted assuming the economic model would be good for 15 years and applying a population projection. Costs expended by social insurers without discounting were counted as budgets.

**Results:**

Annual budget impacts of mass screening compared with do-nothing scenario were calculated as ¥79–¥−1,067 million for dipstick test only, ¥2,505–¥9,235 million for serum Cr assay only and ¥2,517–¥9,251 million for the use of both during a 15-year period. Annual budget impacts associated with the reforms were calculated as ¥975–¥4,129 million for mandating serum Cr assay in addition to the currently used mandatory dipstick test, and ¥963–¥4,113 million for mandating serum Cr assay only and abandoning dipstick test.

**Conclusions:**

Estimated values associated with the reform from ¥963–¥4,129 million per year over 15 years are considerable amounts of money under limited resources. The most impressive finding of this study is the decreasing additional expenditures in dipstick test only scenario. This suggests that current policy which mandates dipstick test only would contain medical care expenditure.

## Introduction

A consensus has been established that chronic kidney disease (CKD) is a worldwide public health problem [1, 2]. The effectiveness of its early detection and treatment to prevent progression to end-stage renal disease (ESRD) and premature death from cardiovascular disease has become widely accepted [3], while the strategy of its screening is still under debate [4]. Whereas high-risk strategies such as routine screening for diabetes patients and as a part of initial evaluation of hypertension patients are pursued in Western countries [5, 6], some argue that population strategies, such as mass screening, could be adopted in Asian countries where CKD prevalence is high [7].

Japan has a long history of mass screening programme for kidney diseases targeting school children and adults since the 1970s. Both urinalysis and measurement of serum creatinine (Cr) level have been mandated to detect glomerulonephritis in annual health checkup provided by workplace and community for adults aged ≥40-year old since 1992 [8]. However, glomerulonephritis was replaced by diabetic nephropathy as the leading cause of ESRD in 1998, and the focus of mass screening policy for adults was shifted to the control of lifestyle-related diseases. In 2008, the Japanese government launched a programme, specific health checkup (SHC) and Specific Counselling Guidance, focusing on metabolic syndrome to control lifestyle-related diseases, targeting all adults between the ages of 40 and 74 years [9]. This is a combined programme of mass screening followed by health education or referral to physicians. During the process of this development of SHC, different types of screening test for kidney diseases were discussed in the health policy arena [10]. Abandonment of dipstick test to check proteinuria was initially proposed by the Ministry of Health, Labour and Welfare, which was opposed by nephrologists who emphasised the significance of CKD. As a consequence, serum Cr assay was alternatively dropped and dipstick test remained in the list of mandatory test items [11]. From the viewpoint of CKD control, the current SHC and Specific Counselling Guidance are not adequate. Therefore, to present evidence regarding CKD screening test for the revision of SHC, which was due in 5 years from its start in 2008, the Japanese Society of Nephrology set up the Task Force for the Validation of Urine Examination as a Universal Screening. Since cost-effectiveness analysis provides crucial information for organising public health programmes such as mass screening, the task force conducted an economic evaluation as a part of their mission, which had been published elsewhere [12]. It concludes that the current policy which mandates dipstick test only is cost-effective, while a policy that mandates serum Cr assay is also cost-effective.

**Fig. 1:**
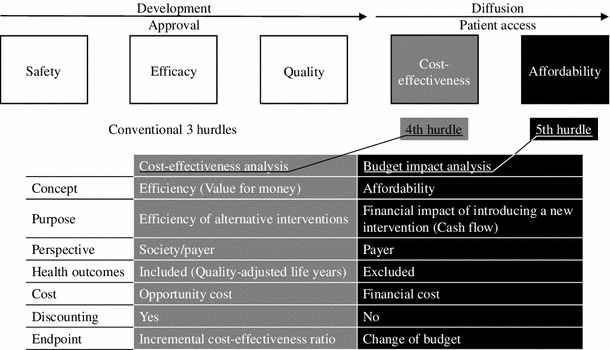
In addition to conventional three hurdles for approval through development phase, two modern hurdles for patient access through diffusion phase are widely recognised these years: 4th hurdle for cost-effectiveness and 5th hurdle for affordability. These hurdles are appraised by cost-effectiveness analysis and budget impact analysis, respectively. Cost-effectiveness analysis concerns efficiency of resources use based on the valuations of cost and effectiveness at the same time comparing technical alternatives, while budget impact analysis concerns affordability of the government or the third party payer by demonstrating changes of cash flows as a result of making an intervention accessible for the population

However, it is said that there are five hurdles to overcome in the nationwide application of health intervention: quality, safety, efficacy, cost-effectiveness and affordability (Fig. 1) [13, 14]. Among these hurdles, ‘cost-effective’ in the economic evaluation framework means that it is acceptable for the society to sacrifice the total value of cumulative costs with discount over the time horizon to gain additional health outcomes brought by the suggested public health programme, whereas it does not directly mean affordability that the government or the third party payer such as social insurers are able to expend required cash to implement the policy. Prevention including mass screening always accompanies costs in advance and effectiveness in the future, which instantly raises a question about its impact on health care financing over time. This paper aims to examine the fifth hurdle, that is, affordability of CKD mass screening test under Japan’s health system by estimating its impact on public health care expenditure [15]. The results would have implications for CKD screening programmes not only in Japan but also for other populations with high prevalence of CKD such as Asian countries [16, 17].

## Methods

We conducted a budget impact analysis of CKD screening test in SHC based on our previous economic model reporting cost-effectiveness [12]. As shown in Fig. 1, the budget impact analysis is to demonstrate budget changes in terms of cash flows, in which payer’s perspective is always taken; health outcomes are excluded; and financial costs are included.

As the summary of the economic model constructed in our previous cost-effectiveness analysis is shown in Table 1, it evaluated two reform policy options based on the economic model comparing do-nothing scenario with dipstick test only, serum Cr assay only, and both. The two policies were: mandate the use of serum Cr assay in addition to the current dipstick test (Policy 1); or mandate the use of serum Cr assay only and abandon dipstick test (Policy 2). Policy 1 meant that the current SHC practice, which was a mandatory 100 % use of dipstick test with 60 % use of serum Cr assay at discretion, would become a mandatory 100 % use of both dipstick test and serum Cr assay; while Policy 2 meant that the current practice would switch to the mandatory 100 % use of serum Cr assay and no use (0 %) of dipstick test. The latter assumption was made by the change in diagnosis criterion of diabetes [18], in which a blood test to check the level of haemoglobin A1c instead of a dipstick test to check urinary sugar level had become pivotal. And the model estimator comparing do-nothing scenario with dipstick test only scenario reflected the choice of continuing the current policy. Our budget impact analysis evaluated these policy options.

**Table 1:** Summary of cost-effectiveness of chronic kidney disease (CKD) screening test in Japan

*Objective* The study aims to assess the cost-effectiveness of population strategy, i.e. mass screening, for CKD control and Japan’s health checkup reform
*Methods* Cost-effectiveness analysis was carried out to compare test modalities in the context of reforming Japan’s mandatory annual health checkup for adults. A decision tree and Markov model with societal perspective were constructed to compare dipstick test to check proteinuria only, serum creatinine (Cr) assay only, or both
*Results* Number of screened patients and incremental cost-effectiveness ratios (ICERs) of mass screening compared with do-nothing were calculated as 832 patients out of 100,000 participants and ¥1,139,399/QALY (US $12,660/QALY) for dipstick test only; 3,448 patients and ¥8,122,492/QALY (US $90,250/QALY) for serum Cr assay only; and 3,898 patients and ¥8,235,431/QALY (US $91,505/QALY) for both. Number of additionally screened patients and ICERs associated with the reform were calculated as 1,061 (3,898 from 2,837) patients out of 100,000 participants and ¥9,325,663/QALY (US $103,618/QALY) for mandating serum Cr assay in addition to the currently used mandatory dipstick test (Policy 1), and 611 (3,448 from 2,837) patients ¥9,001,414/QALY (US $100,016/QALY) for mandating serum Cr assay and applying dipstick test at discretion (Policy 2). The decrease of new haemodialysis patients compared with do-nothing in the fifth year and tenth year were estimated as 0.293 %/1.128 % for dipstick test only, 5.092 %/4.380 % for serum Cr assay only, and 5.094 %/4.380 % for both. The decrease of new haemodialysis patients associated with the reform was 1.249 %/1.346 % for Policy 1 and 1.251 %/1.346 % for Policy 2
*Conclusions* Taking a threshold to judge cost-effectiveness according to World Health Organization’s recommendation, i.e. three times gross domestic product per capita of ¥11.5 million/QALY (US $128 thousand/QALY), a policy that mandates serum Cr assay is cost-effective. The choice of continuing the current policy which mandates dipstick test only is also cost-effective. Results suggest that a population strategy for CKD detection such as mass screening using dipstick test and/or serum Cr assay can be justified as an efficient use of health care resources in a population with high prevalence of the disease

*Source* Kondo et al. [12]

Health care budget impact is defined as a forecast of rates of use (or changes in rates of use) with their consequent short- and medium-term effects on budgets and other resources to help health service managers plan such changes [19]. We took the following three steps in our analysis: (1) the estimation of annual incremental budget per person, (2) the estimation of annual number of adults who would uptake SHC and (3) the estimation of budget impact by combining the results from (1) and (2).

The first step (1) was implemented on our economic model assuming that the annual economic model would be good for 15 years (Table 2). It included costs borne by adults and social insurers from the societal perspective, while costs of sectors other than health and productivity losses were uncounted. Costs expended by social insurers without discounting were counted as budgets. Costs for screening were fully borne by social insurers, and costs for further detailed examination and treatment at health facilities were 70 % reimbursed except in case of dialysis. Fixed co-payment for dialysis patients, ¥10,000 (US$100, US$1 =¥100) per month, was subtracted from the total cost. Assumed annual budgets per person are shown in Table 2.

**Table 2:** Assumptions for budget impact analysis

1. The annual economic model is good for 15 years		
2. Annual budgets per person (costs in the economic model [12])		
Screening		
Dipstick test only	¥ 267 (¥267)	
Serum Cr assay only	¥138 (¥138)	
Dipstick test and serum Cr assay	¥342 (¥342)	
Detailed examination at clinic or hospital	¥17,500 (¥25,000)	
CKD treatment		
Stage 1	¥84,000 (¥120,000)	
Stage 2	¥102,900 (¥147,000)	
Stage 3	¥235,900 (¥337,000)	
Stage 4	¥555,100 (¥793,000)	
Stage 5	¥691,600 (¥988,000)	
ESRD treatment	¥5,880,000 (¥6,000,000)	
Heart attack treatment		
1st year	¥1,946,000 (¥2,780,000)	
2nd year and after	¥125,300 (¥179,000)	
Stroke treatment		
1st year	¥700,000 (¥1,000,000)	
2nd year and after	¥125,300 (¥179,000)	
3. A population projection for Japan [17] is used and sex and age structure is applied for the annual economic model		
4. The uptake of SHC is fixed at 41.3 % for 15 years [18]		

In the second step (2), we used a population projection for Japan [20], and sex and age structure was applied to our annual economic model. We assumed that the uptake of SHC was fixed at 41.3 % for 15 years [21]. In the third step (3), estimated annual incremental budgets per person were multiplied by estimated annual number of adults who would uptake SHC.

## Results

**Fig. 2:**
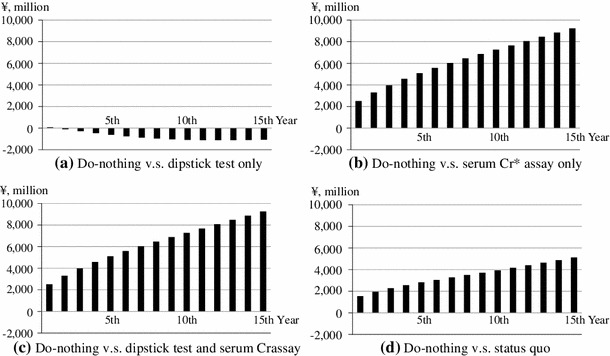
*Black bars* depict annual budget impacts of mass screening compared with do-nothing scenario. Negative budget impacts on (**a**) imply that the continuation of current policy which mandates dipstick test only would contain medical care expenditure. **a** Do-nothing versus dipstick test only. **b** Do-nothing versus serum Cr assay only. **c** Do-nothing versus dipstick test and serum Cr assay. **d** Do-nothing versus status quo. *Cr* creatinine

Table 3 shows the model estimators of budget impact. Compared with do-nothing scenario, total additional expenditure of dipstick test only decrease from ¥79 million (US$0.79 million) in the first year (2012) to ¥−1,067 million (US$−10.67 million) in the fifteenth year (2026); those of serum Cr assay only increase from ¥2,505 million (US$25.05 million) to ¥9,235 million (US$92.35 million); those of both dipstick test and serum Cr assay increase from ¥2,517 million (US$25.17 million) to ¥9,251 million (US$92.51 million); and those of status quo increase from ¥1,542 million (US$15.42 million) to ¥5,122 million (US$51.22 million). These estimators are also shown in Fig. 2. The breakdown of additional expenditures for screening and curative care is also reported in Table 3. Additional expenditures for screening are almost constant: ¥16 million (US$0.16 million) for dipstick test only, ¥8 million (US$0.08 million) for serum Cr assay only, ¥20 million (US$0.2 million) for dipstick test and serum Cr assay, and ¥18 million (US$0.18 million) for status quo. Decreases or increases during the 15 years are attributable to the changes in additional expenditure for curative care.

**Table 3:** Model estimators of budget impact

Year	Budget impact: total additional expenditure (¥, million)				Additional expenditure for screening (¥, million)				Additional expenditure for curative care (¥, million)			
	Dipstick test only	Serum Cr assay only	Dipstick test and serum Cr assay	Status quo	Dipstick test only	Serum Cr assay only	Dipstick test and serum Cr assay	Status quo	Dipstick test only	Serum Cr assay only	Dipstick test and serum Cr assay	Status quo
1st (2012)	79	2,505	2,517	1,542	16	8	20	18	64	2,497	2,497	1,524
2nd (2013)	−96	3,295	3,308	1,946	16	8	20	18	−112	3,287	3,288	1,928
3rd (2014)	−278	3,972	3,985	2,280	16	8	20	18	−294	3,964	3,965	2,262
4th (2015)	−454	4,561	4,574	2,563	16	8	20	18	−470	4,553	4,554	2,545
5th (2016)	−615	5,089	5,103	2,815	16	8	20	18	−631	5,081	5,083	2,797
6th (2017)	−755	5,572	5,586	3,049	16	8	20	18	−771	5,564	5,566	3,031
7th (2018)	−872	6,025	6,039	3,274	16	8	20	18	−887	6,017	6,019	3,256
8th (2019)	−964	6,453	6,467	3,494	16	8	20	18	−979	6,445	6,447	3,476
9th (2020)	−1,032	6,861	6,875	3,712	16	8	20	18	−1,048	6,853	6,855	3,693
10th (2021)	−1,079	7,261	7,275	3,933	16	8	20	18	−1,094	7,252	7,255	3,915
11th (2022)	−1,105	7,660	7,675	4,162	16	8	20	18	−1,120	7,652	7,655	4,144
12th (2023)	−1,114	8,060	8,076	4,399	16	8	20	18	−1,129	8,052	8,056	4,380
13th (2024)	−1,109	8,456	8,472	4,638	16	8	20	18	−1,124	8,448	8,452	4,620
14th (2025)	−1,092	8,845	8,861	4,878	16	8	20	18	−1,108	8,837	8,841	4,860
15th (2026)	−1,067	9,235	9,251	5,122	16	8	20	18	−1,083	9,227	9,231	5,104

*Cr* creatinine

**Fig. 3:**
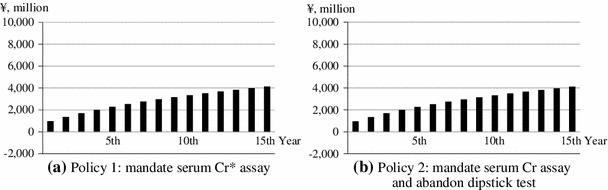
*Black bars* depict annual budget impacts associated with suggested mass screening policy reforms which mandate the use of serum Cr assay. Positive budget impacts on both panels imply that the reforms would result in the increase of medical care expenditure. **a** *Policy 1* mandate serum Cr assay. **b** *Policy 2* mandate serum Cr assay and abandon dipstick test. *Cr* creatinine

Table 4 shows the results of budget impact analysis in the same way focusing on the two policy options. Compared with status quo, the budget impacts as total additional expenditure of Policy 1 which requires serum Cr assay increase from ¥975 million (US$9.75 million) in the first year (2012) to ¥4,129 million (US$41.29 million) in the fifteenth year (2026); and those of Policy 2 which requires serum Cr assay and abandons dipstick test increase from ¥963 million (US$9.63 million) to ¥4,113 million (US$41.13 million). These are drawn in Fig. 3 as well. Breakdowns of screening and curative care are also reported in Table 4. Additional expenditures for screening are almost constant: ¥2 million (US$0.02 million) for Policy 1, and ¥−10 million (US$−0.1 million) for Policy 2. Increases during the 15 years are attributable to the changes in additional expenditure for curative care.

**Table 4:** Results of budget impact analysis

Year	Budget impact: total additional expenditure (¥, million)		Additional expenditure for screening (¥, million)		Additional expenditure for curative care (¥, million)	
	Policy 1: mandate serum Cr assay	Policy 2: mandate serum Cr assay and abandon dipstick test	Policy 1: mandate serum Cr assay	Policy 2: mandate serum Cr assay and abandon dipstick test	Policy 1: mandate serum Cr assay	Policy 2: mandate serum Cr assay and abandon dipstick test
1st (2012)	975	963	2	−10	973	973
2nd (2013)	1,362	1,349	2	−10	1,360	1,359
3rd (2014)	1,705	1,692	2	−10	1,704	1,702
4th (2015)	2,011	1,998	2	−10	2,010	2,008
5th (2016)	2,287	2,274	2	−10	2,285	2,284
6th (2017)	2,537	2,523	2	−10	2,535	2,533
7th (2018)	2,765	2,751	2	−10	2,763	2,761
8th (2019)	2,973	2,958	2	−10	2,971	2,969
9th (2020)	3,164	3,149	2	−10	3,162	3,159
10th (2021)	3,342	3,328	2	−10	3,341	3,338
11th (2022)	3,513	3,498	2	−10	3,511	3,508
12th (2023)	3,677	3,662	2	−10	3,675	3,672
13th (2024)	3,833	3,818	2	−10	3,832	3,828
14th (2025)	3,983	3,967	2	−10	3,981	3,977
15th (2026)	4,129	4,113	2	−10	4,127	4,123

*Cr* creatinine

## Discussion

We estimate the budget impacts of CKD screening test in SHC, of which use has been found cost-effective elsewhere [12]. With regard to two reform policy options: mandate serum Cr assay in addition to the dipstick test (Policy 1), and mandate serum Cr assay and abandon dipstick test (Policy 2), both positive and increasing budget impacts are found in the fifteen-year time frame. Although there is no established rule for interpreting the results of budget impact analysis, estimated values of ¥963 million (US$9.63 million) to ¥4,129 million (US$41.29 million) per year over fifteen years are considerable amounts of money of limited resources. These amount to 0.0026 to 0.011 % of national medical care expenditure in 2010 [22], and 0.068 and 0.29 % of the annual increase between 2009 and 2010, ¥1,413,500 million (US$14,135 million), respectively. Our case study exemplifies a situation where budgetary constraints, or affordability, matters to the use of cost-effective interventions which have been judged as worth using according to social willingness to pay for new intervention.

The most impressive finding of this study, however, is the decreasing additional expenditures of dipstick test only scenario, which become negative in just its second year. This suggests that the mandatory dipstick test under current practice would contain medical care expenditure, i.e. ‘decreasing annual national medical costs’. In other words, this is a valuable evidence that prevention saves life as well as money. And requiring dipstick test instead of serum Cr assay as a mandatory test item in SHC in 2008 may have been a sensible choice.

Due caution is needed to interpret the results of our budget impact analysis, since they depend on crucial assumptions. Positive budget impacts are found to be attributable to additional expenditure for curative care; however, for example, the analysis does not take medical advancement or health system development into account. In the coming 15 years, innovative therapeutic agents to prevent progression to ESRD are expected [23–26], and community-based CKD control intervention under collaboration between general practitioners and nephrologists is under study [27]. More prevention of ESRD should bring significant reduction in budget impact, since treatment of ESRD is most costly. With regard to the mass screening test, other tests such as microalbuminuria or cystatin C could be an option in the middle to long run [24], which would fundamentally change the background of this analysis.

In the policy arena, the revision of SHC after its first five-year period was made in 2012, in which the continuation of current policy was chosen. And our study is in accord with keeping dipstick test in the mandatory test list. Further economic evaluation incorporating medical advancement or health system development is necessary for the future development of SHC and the next revision of CKD mass screening.
